# New perspectives for colorectal cancer

**DOI:** 10.18632/oncotarget.18448

**Published:** 2017-06-13

**Authors:** Alberto Puccini, Heinz-Josef Lenz

**Affiliations:** Division of Medical Oncology, Norris Comprehensive Cancer Center, Keck School of Medicine, University of Southern California, Los Angeles, CA, USA

**Keywords:** colorectal cancer, microsatellite instability, BRCA2, EGFR, NTRK

Colorectal cancers that arise from microsatellite instability (MSI) pathway, caused by deficient DNA mismatch repair (dMMR) activity, show a specific phenotype that is characterized by predilection for right side, BRAF mutation (50%), female sex, abundant lymphocyte infiltration, poor differentiation, overall better prognosis and a worse response to 5-FU. Its prevalence is higher in early stage (20% stage I-II; 12% stage III) than advanced disease (4% stage IV) [1]. Moreover, MSI tumors have been demonstrated to carry a much higher load of mutations in comparison to MSS [2]. This is believed to be one of the reasons why MSI tumors better benefit and response to immunotherapy than microsatellite stable (MSS).

In this current issue, Deihimi and coworkers [3] sought if MSI status can lead to mutations in BRCA2, EGFR and NTRK in colorectal cancer. They elegantly have demonstrated a significant higher mutation rates both in a discovery cohort of 584 patients (26 MSI-high and 558 non-MSI-H from Caris Life Science database) and in a second cohort of 1104 patients (COSMIC and TCGA data set). BRCA2 mutation in MSI-H was 38% versus 6% of non-MSI CRCs (*p* < 0.001); EGFR was mutated in 45.5% of MSH2/MLH1-mutant and 6.5% of non-MSH2/MLH1-mutant tumors (*p* < 0.001). NTRKs mutations were discovered in 40% of MSH2/MLH1-mutant CRCs versus 16% of non-MSI-H (*p* = 0.0003).

The rationale to test these three families of genes is sound and clinically relevant: a) BRCA2 is an important element for homologous recombination (HR), that is influenced and modulated by mismatch repair proteins, such as MSH2 and MLH1. In addition, BRCA2 is rich of microsatellites that make it a vulnerable target in dMMR tumors; b) overexpression of EGFR is a well-known cancer driver alteration: activating mutations that occur in tyrosine kinase domain can be inhibited by tyrosine-kinase inhibitor (TKI), as already implemented in NSCLC. Hence, looking for new targetable mutations in CRC is crucial; c) finally, NTRK family genes encode for receptor tyrosine kinases involved in physiology of development of nervous system through activation by neurotrophins [4]. Rearrangements (especially fusions) of NTRK genes has been showed to be cancer drivers through activation of downstream signaling of MAPK-ERK, and PTEN-PI3K-AKT. Even if NTRK mutations are rare, they can lead to cancer and progression in different type of cancer, including CRC, where it has been originally discovered [5]. More importantly, the clinical significance of these selected genes is the fact that they are easily druggable either because there are already available compounds (PARP inhibitors for BRCA2-mut and TKI for activating EGFR-mut) or under extensive study in clinical trials (NTRK inhibitors) [6].

This research has some limitations. The first is the heterogeneity of the two cohorts, one from Caris Lifes Sciences and the other from the Catalog of Somatic Mutations in Cancer (COSMIC) database and The Cancer Genome Atlas data set. In addition, in the second cohort the MSI status was missing, but authors came up with a reasonable solution: they sought mutations in MLH1 and MSH2, since inactivation of MLH1 and MSH2 account for over 90% of dMMR cases. Nevertheless, they could have missed those MSI tumors caused by MLH1 promoter methylation, that may account for the majority of MSI CRCs [7]. Moreover, the unavailability of allele frequency particularly for BRCA2, and the lack of the protein expression level of EGFR make it harder to draft conclusions about the real clinical implications of these mutations, whereas the authors used a functional prediction modeling to reveal deleterious mutations. Of note, it is not currently the standard to demonstrate protein expression in EGFR-mutant NSCLC (nor is it feasible, e.g. in liquid biopsy), and while some mutations in EGFR are predicted to damage the protein, the authors predict about 15% among those they found mutated in mismatch-repair deficient colorectal tumors may activate the EGFR kinase domain and some are known to do so.

The higher rates of mutations in these genes may be expected because of the intrinsic carcinogenesis pathway of dMMR tumors. Nevertheless, in order to find new therapeutic option for patients, searching for mutations in well-known genes that can be actionable, may have immediately repercussion on clinical practice and therefore changing therapeutic strategies for CRC patients: BRCA2 mutated cancer can benefit from PARP-inhibitors, as recently showed in ovarian cancer [8]. EGFR mutations that occur in TK domain may be activating mutations and then can be susceptible to EGFR-inhibitors, such as small molecules like erlotinib, gefinitib or osimertinib in NSCLC and antibodies like cetuximab and panitumumab. Finally, NTRK inhibitors (like entrectinib and LOXO-101) have already shown promising results in early phase trials and may represent a future option for the small subset of CRC patients harboring NTRK alterations (fusion or mutation).

Personalized medicine has been already implemented in daily practice in the last decade for cancer patients, and it will guide medical oncologists to choose treatment that best tailored to the specific patient’s characteristics. This research moves a tremendous step forward in this direction and it suggests to going further to address if these mutations are functional significant and if they are targetable. This will increase our therapeutic options even if for a small subset of CRC patients.
